# Hormonal and structural transformations in the caregiver's brain: Examining themes in parental neurobiology

**DOI:** 10.3934/Neuroscience.2025030

**Published:** 2025-11-26

**Authors:** Elaine K. Feller, Erin N. McGinity, Patrick William Cafferty

**Affiliations:** Department of Biology, Emory University, 1510 Clifton Road NE, Atlanta, GA 30322, USA

**Keywords:** parental neurobiology, medial preoptic area, estrogen, progesterone, testosterone, maternal behavior, paternal behavior, postpartum depression

## Abstract

When becoming a parent, caregivers undergo complex, and sometimes permanent, neurobiological alterations, and this area of neurobiology has been extensively studied for decades. Due to ethical concerns and experimental limitations, the first parental neurobiology experiments were exclusively performed using rodent animal model systems, such as mice, rats, and voles. More recent technological advancements, such as the functional MRI (fMRI) scan, have become widely adopted and led to great insight into the impact of parenting on human neurobiology. In this thematic literature review, we present key studies that provide insight into the relationship of pregnancy and parturition on maternal caregiving behavior and the relationship of postpartum on all parents. First, we examine the relationship of endocrine hormones such as estrogen, progesterone, oxytocin, and testosterone with the neurobiological development of a parent. Next, we describe the significant transformation of subcortical maternal circuit components that occur during pregnancy, and the changes in the volume of grey and white matter generated during the postpartum. These brain structure alterations contribute to the development of parental nurturing behaviors.

## 1. Introduction

Since the emergence of parental neuroscience approximately 100 years ago, the experience of becoming a parent has come to be recognized as a true neurobiological transformation [1]. The physical and hormonal stimuli associated with parenthood trigger both temporary and lasting changes in the mammalian brain, thereby activating networks that previously sat latent, altering the way hormones are interpreted, and modifying the physical volumes of key brain regions [2]–[4]. These changes arise in an evolutionary context as mammals give birth to relatively undeveloped, helpless young, and thus parents must invest a significant share of their finite time and energy to attend to their offspring [3].

In many mammals, nurturing behaviors, and the underlying brain alterations that contribute to their development, are only activated once an individual becomes a parent. Nurturing behaviors are rarely a “default setting”, thereby preventing the misallocation of resources that could otherwise be directed towards self-maintenance or courtship and mating [5]. Relatedly, parental habits become stably ingrained and resistant to hormonal fluctuations to ensure that caregivers remain dedicated to their young throughout their upbringing under ever changing conditions [6].

Early studies in the field of parental neuroscience were limited by cultural norms and the available experimental technologies [1]. These studies specifically examined the hormonal changes that took place in mothers while parenting within a nuclear family, with little attention paid to the impact of parenting upon fathers and other caregivers within diverse family structures [7],[8]. More recent studies of fathers and other caregivers have revealed that the neurobiological changes associated with parenthood may be triggered by stimuli such as scent, physical touch, and social reciprocity, and not only pregnancy, childbirth, or lactation [9].

The development and implementation of non-invasive imaging techniques such as functional magnetic resonance imaging (fMRI) have identified new brain regions that are involved in parenthood, thus providing further insight into human neurobiology. This review will examine key neurobiological developments which elucidate the impact of parenthood on endocrine hormone signaling and brain structures in diverse caregivers, and how these alterations shape parenting behaviors.

## 2. Hormonal control of parental nurturing behavior

### 2.1. Parenting impacts hormonal levels and sensitivity within the brain and shapes nurturing behavior in rodent model systems

A wealth of studies published in the 1970s explored the question of how parenting changes hormonal levels and sensitivity in the rodent brain [10]–[14]. Scientists explored this question using rodents such as the Norway rat (*Rattus norvegicus*) and California mouse (*Peromyscus californicus*), as these animal models exhibit similar maternal caregiving patterns to humans [15],[16], and the measurement of hormones and hormone receptors in the brain required invasive experimental techniques, including the removal and chemical processing of vital neural tissues. Furthermore, insights gained from studies of rodent model systems served as an important starting point and source of hypotheses for researchers studying humans.

In some rodent species, mothers immediately nurture their pups through approach, licking, grooming, and retrieving after giving birth (parturition), while these same caregiving behaviors emerge more gradually in nulliparous females housed in close quarters with pups [10],[17]. This rapid maternal response toward newborn offspring is primed by hormones associated with pregnancy and parturition [6] that act on the medial preoptic area (mPOA), which is a vital brain region for caregiving behaviors [18]. In addition, hormones act on other regions of the brain, such as the adjacent, functionally linked ventral bed nucleus of the striatum terminalis, the medial amygdala, and the hippocampus to regulate caregiving behaviors [19]. While these regions are commonly known as the “anxiety center”, “fear center”, and “memory center”, respectively, these oversimplified shorthands risk drawing overly broad conclusions about hormonal effects that fail to account for the core brain regions' more complex and interconnected roles [19]. This review will first examine changes to the mPOA that occur during parenting, followed by an exploration of alterations generated in other relevant brain regions.

### 2.2. Maternal caregiving behavior is directly mediated by hormones within the mPOA

The field of parental neurobiology was sharply focused by work from Michael Numan and colleagues, who discovered the necessary and specific role of the mPOA in maternal caregiving behaviors [20]. Guided by this work, subsequent studies have shown that in mammals, the gradual increase of estrogen and progesterone during pregnancy, followed by their sharp drop at birth, collectively influences maternal caregiving behaviors by acting on this key brain region. Numan et al. [20] mimicked the rise of estrogen during pregnancy by implanting estradiol benzoate into the mPOA of nulliparous female rats and observed a shorter delay (latency) before maternal behaviors were first exhibited compared to control animals implanted with cholesterol in the mPOA or in rats with estradiol benzoate implanted in other brain regions. Relatedly, the loss of estrogen receptor-α (ERα) activity impairs the maternal care of pups, as revealed by examinations of ERα knock-out mice [21], mice with ERα silenced in the preoptic area by RNA interference [22], and mPOA-specific ERα knockout mice [23]. In addition, Fang et al. [24] demonstrated that the optogenetic activation of ERα-expressing neurons in the mPOA promoted pup gathering by female mice, while an inhibition of these neurons led to a delay in pup retrieval, which suggests that estrogen might exert transcriptional effects to lower the activation threshold of ERα-expressing neurons and increase their probability of firing.

Progesterone exhibits a synergistic effect with estradiol [10],[25], and progesterone blood levels must rise during pregnancy [26] and fall during birth to promote maternal caregiving behaviors [27]. Consistent with a loss of ERα signaling in the mPOA, mPOA-specific knockout of the progesterone receptor has been shown to inhibit maternal pup retrieval and nursing behaviors [23].

Additionally, oxytocin, vasopressin, and prolactin contribute to the development of maternal caregiving behaviors, though the interpretation of loss-of-function experiments for these signaling molecules is challenging due to their roles in other reproductive behaviors, including parturition and milk production, and the interconnected nature of their signaling pathways [3]. Consistent with a role for oxytocin to promote maternal caregiving behaviors, Tsuneoka et al. [28] observed decreased pup retrieval by virgin female Oxtr-knockout mice under stressful conditions, and this stress vulnerability was inhibited in Oxtr and vasopressin receptor 1a (Avpr1a) double-knockout mice. However, in a study by Hidema et al. [29], lactation and prolactin production were inhibited but the pup-retrieval behavior was unaffected in mPOA-specific Otxr knockout mice. Similar to numerous studies in the field of parental neurobiology, the work of Hidema et al. [29] was limited by the narrow metrics used to quantify maternal behaviors. The main conclusion of this study—that mPOA-specific Otxr knockout mice exhibited decreased milk production but did not affect maternal caregiving behaviors—was solely based on measurements of pup retrieval, time spent crouching in a lactation stance, and pup body weight. Since the first two metrics of maternal behaviors did not differ significantly from controls, Hidema et al. [29] primarily attributed the low pup body weight to reduced lactation. While Hidema et al. [29] suggested deficits in unmeasured aspects of maternal care, such as difficulty sustaining attentiveness, may have exacerbated the low body weight of pups, this hypothesis was not directly tested in their study. To precisely determine how and to what extent oxytocin, vasopressin, and prolactin contribute to caregiving behaviors in all caregiver types, future studies must report a standardized, comprehensive set of behavioral metrics. This would allow for a clearer understanding of how various hormone treatments affect caregiving and significantly improve the ability to draw comparisons between studies. We recommend that future work include data on pup retrieval, nest building, lactation posturing, licking and grooming, and huddling. Quantifying both the latency to first exhibit these behaviors and the total duration of each activity will provide the most complete picture of parental caregiving. By listing the diverse measures used in representative studies, Table 1 makes clear the lack of standardization in quantifying nurturing behaviors within current parental neurobiological research.

### 2.3. Paternal and foster parent caregiving behavior is impacted by endocrine hormones

Although pregnancy grants mothers a head start in the parental response, in a minority of mammalian species, caregiving activities are also eventually observed in fathers and foster parents that never experienced pregnancy. Among rodents, paternal caregiving greatly varies among species, with some species being biparental in nature, such as the prairie vole (Microtus ochrogaster) and California mouse, and other species who do not show paternal behaviors in the wild but can be induced to behave paternally under experimental conditions, including the Norway rat [30]. The delay in caregiving behaviors or “latency”, varies from rodent species to species, from mere seconds in inbred mice to several days in rats [23],[31].

Hormonal events may play some part in establishing paternal behaviors, as blood estrogen rises in male California mice shortly after the birth of their litter [32]. While the paternal increase in blood estrogen is accompanied by a drop in progesterone, these hormonal fluctuations are far less dramatic than those recorded in the maternal blood [33]. Some evidence suggests that this increase in circulating estrogen is not required for paternal caregiving, as the surgical castration of male dwarf hamsters by Hume and Wynne-Edwards [34] reduced the blood estradiol concentration but did not affect paternal caregiving. However, paternal caregiving may have persisted in castrated animals due to the central production of estradiol. Consistent with this hypothesis, when Trainor and Marler [35] treated castrated California mice with both testosterone and an inhibitor of the aromatase enzyme that converts testosterone to estradiol, they observed a reduction in huddling and pup grooming behaviors compared to castrated control animals. In addition, recent work by Duarte-Guterman et al. [36] using mice revealed a correlation between the amount of paternal experience and the number of aromatase-expressing cells in several brain regions. This evidence suggests that estrogen signaling plays a role in the promotion of paternal caregiving behaviors, as had previously been demonstrated in mothers.

**Table 1.** Methods of quantifying caregiving behavior vary widely between studies.

Species	Caregiver	Treatment	Measure	Units	Conclusion	Reference
Rat	Nulliparous foster mothers	Estrogen, progesterone, prolactin replacement	Latency to exhibit all five: • Pup retrieval • Nest building • Lactation posture • Licking and grooming • Huddling	Hours	Hormone treatment hastens onset of maternal behavior	Moltz et al. (1970) [10]
Rat	Nulliparous foster mothers	Estrogen and progesterone replacement	Latency to exhibit both: • Pup retrieval • Sustained lactation posture	Days	Progesterone delays estrogen-induced maternal behavior	Siegel and Rosenblat (1975) [12]
Mouse	Nulliparous foster mothers	siRNA silencing of estrogen receptor-α	Latency to retrieve pups; Time spent licking and grooming; Time spent nursing	Seconds	Silencing of estrogen receptor-α impairs maternal behavior	Ribeiro et al. (2012) [22]
Mouse	Mothers; Nulliparous foster mothers	Knockout of oxytocin receptor in the mPOA	Pup body weight; Pup stomach weight; Latency to sniff pups; Time licking pups; Neural activation	Grams; Seconds; c-fos positive cells per mm^2^	Oxytocin receptors are not essential for maternal behavior	Hidema et al. (2024) [29]
Dwarf hamster	Fathers; Foster fathers	Castration	Latency to contact pups; Latency to pick up pups; Latency to retrieve pups back to nest	Seconds	Castration does not impair paternal behavior	Hume and Wynne-Edwards (2005) [34]
California mouse	Fathers; Foster fathers	Castration with or without testosterone replacement	Time huddling; Time licking and grooming	Seconds	Castration reduces paternal behavior	Trainor and Marler (2001) [35]
Mandarin vole	Fathers; Paternally experienced foster fathers; Virgin foster fathers	Injection of oxytocin receptor antagonist	Latency to approach pups; Latency to sniff pups; Latency to retrieve pups; Time licking and grooming; Time in lactation posture	Seconds	New fathers exhibit more prompt nurturing behavior. Oxytocin receptor antagonist reduces paternal behavior	Yuan et al. (2019) [37]

Note: Table 1 highlights the inconsistent use of measures to quantify nurturing behavior in a representative selection of maternal behavior studies elaborated upon in section 2.2, and studies conducted in males, further addressed in section 2.3.

The caregiving behaviors that gradually emerge in some fathers have been shown to be directed by the action of oxytocin in the mPOA [37]. For instance, Yuan et al. [37] revealed that the oxytocin receptor expression is elevated in the mPOA of mandarin vole fathers compared to virgin males approximately ten days after the birth of their young. Conversely, Perea-Rodriguez et al. [38] found that the oxytocin receptor mRNA levels were not significantly different in the mPOA of California mouse fathers compared to controls. These contradictory findings raise doubts about the cross-species conservation of hormonal mechanisms. However, Perea-Rodriguez et al. [38] measured the oxytocin receptor mRNA levels two to four days postpartum. Possibly, not enough time had elapsed before the oxytocin receptor mRNA measurements for the California mouse fathers to become sensitized by the scent, sound, and touch of their pups. Pal et al. [3] reviewed studies that compared changes in hormone receptor levels across species, but the precise developmental stage at which these measurements were taken often differed between studies, or the timing of measurement was not reported. The absence of consistent use and reporting of the timing of measurements is a broad challenge in the field of parental neuroscience, thus making comparisons between different studies and across species challenging.

Further research into to the hormonal mechanisms of paternal behaviors is needed to counterbalance the extensive literature on maternal neurobiology. Additionally, the hormonal basis of cooperative parenting—in both same-sex and opposite-sex pairs—remains critically underexplored. Expanding the research scope to include diverse family structures will better align the literature with the full range of parenting observed in nature, including among humans.

### 2.4. Motivation for and maintenance of long-term parental care

Once activated, the mPOA may activate a reward circuit to support long-term parental motivation. The mPOA contains neural projections to the ventral tegmental area, which is a component of the mesolimbic dopamine reward system. Stolzenberg and Rissman [39] have proposed that the spikes in oxytocin level and mPOA activation associated with the early days of parenthood may tag offspring as a social reward, while Numan [40] suggested that the dopamine reward system is sufficient to sustain parental responsiveness independently of hormonal action.

Once established, parental behaviors become resistant to hormonal fluctuations. For example, female rats exhibit significantly less licking and grooming compared to control animals when their ovaries were removed within 24 hours of parturition [41]. However, when ovariectomy was delayed to 48–72 hours post-parturition, maternal behaviors could not be eliminated [42]. In fact, when surgery was delayed until after maternal behaviors were established, ovariectomized females were observed to lick their pups more frequently, possibly due to the withdrawal of progesterone [42]. Together, these observations suggest that hormones play a greater role in the establishment, rather than the maintenance, of parental care, and parental behaviors may be maintained over time by the activation of the dopamine reward system.

### 2.5. Resistance to hormonal fluctuations is required for long-term care giving

Resistance to hormonal fluctuations following the establishment of parenting behaviors is important to maintain long-term parental care. For example, progesterone inhibits the onset of maternal behaviors in rats when administered during late pregnancy [12],[14]. However, when progesterone levels rise during lactation, peaking around the tenth day postpartum when maternal behaviors have become established, rats do not abandon their young [13]. In other rodents, progesterone levels return to their regular fluctuations when the estrus cycle resumes, but females have not been observed to cyclically neglect their pups [43]. In addition, progesterone levels steadily rise during subsequent pregnancies, but females often care for past litters while pregnant with future litters, sometimes even simultaneously caring for multiple litters [44]. This may result from a reduction in the mPOA progesterone receptor density brought on by parturition that shields the mPOA from the effect of hormonal fluctuations to preserve parental behavior patterns [31],[42].

Parental behaviors in fathers is resistant to hormonal changes that would cause neglect or infanticide in non-paternal rodents. For example, Hyer et al. [32] demonstrated that estrogen levels in California mouse fathers initially rise after the birth of their litter and subsequently steadily decline to baseline levels at day 16 postpartum. Possibly, paternal care is sustained in these animals due to an increase in estrogen receptor-β (ERβ) expression in key regions of brain structures such as the hippocampus [32], that is, estrogen sensitivity within the brain increases to allow fathers to maintain paternal behaviors despite a return to baseline hormone levels. Similarly, male house mice are aggressive toward young before becoming fathers, and Schneider et al. [33] showed that implanting nonpaternal mice with progesterone increased the likelihood that they would attack pups. However, Schneider et al. [33] were neither able to prompt infanticide nor could they impair established caregiving habits by any observable measure through administration of progesterone to paternal mice. Possibly, progesterone receptors are significantly less abundant in the mPOA of paternal house mice, as has been observed in female rats and in male California mice [31],[38].

In some cases, paternal behaviors can be shielded from the typical effects of testosterone. In virgin males, testosterone typically directs resources towards the mating effort by increasing the mass of reproductive structures such as the seminal vesicles and ventral prostate [45] and, in many rodent species, as well as in humans, the blood testosterone levels are correlated with aggression [46],[47]. However, under certain conditions, high levels of testosterone do not interfere with paternal care. For example, castration of male California mice eliminates pup huddling, while the administration of supplementary testosterone rescues the huddling behavior [35]. Relatedly, the administration of testosterone to Mongolian gerbil fathers also increases the huddling behavior, though gerbils still exhibited aggression towards intruders with further testosterone injections [48]. In addition, testosterone levels are positively correlated with childcare involvement in Filipino men, though not in those individuals genetically predisposed to high levels of the testosterone receptor [49]. These observations may be explained, at least in part, by the increased levels of aromatase in the mPOA of paternal California mice. Restriction of an increased aromatase activity to the mPOA resulted in undetectably low blood estrogen levels [2]. Consequently, the parental control center is site-specifically shielded from the effects of testosterone. Thus, while testosterone is generally negatively correlated with paternal behaviors [50], under certain conditions, the parental control center of the brain can be protected to allow fathers to reliably attend to their young, even as they continue to fight intruders and seek new mates.

In summary, hormonal events associated with motherhood, such as pregnancy, parturition, and lactation, prime the parenting control center of female rodents so that they may exhibit an immediate maternal caregiving response. However, the same parenting control center may be kindled more gradually by prolonged cohabitation with young. Once parental habits are established, the behavior of parents becomes more resistant to hormonal fluctuations compared to that of non-parents. However, this is not to say that parental behaviors become entirely insensitive to hormones. This relative stability allows caregivers to sustain parental behaviors even as they return to other pursuits, such as subsequent pregnancies or territorial defense.

## 3. Structural transformations in the parental brain

### 3.1. Parenthood alters distinct subcortical and cortical structures to facilitate nurturing behavior

The hormonal changes of parenthood alter the brain's neuroplasticity, which results in temporary and long-term changes [51],[52]. Swain [53] has hypothesized that these changes occur in subcortical structures found deep within the brain, including the basal ganglia and related regions such as the striatum, amygdala, hypothalamus, and hippocampus, and in regulatory cortical regions, such as the anterior cingulate, insula medial frontal, and the orbitofrontal cortices. These regions are suggested to change during parenthood as they are associated with emotions, drive, and empathy [53]. Next, we will investigate the structural rearrangements that occur during pregnancy and postpartum within maternal circuits, as well as neural pathways in the brain that regulate maternal behaviors, followed by an examination of grey and white matter volume modifications in specific cortical regions that are generated in response to parenting. Finally, we will examine the functional consequences of these structural changes upon parental caregiving behaviors.

### 3.2. The maternal circuitry includes the mPOA, the prefrontal cortex, amygdala, and nucleus accumbens

As described above, mPOA function is greatly affected by the surge in maternal hormones, in particular estrogen, progesterone, and oxytocin. Some authors, such as Pawluski [54], have described the mPOA as the central hub of a maternal circuit that consists of the mPOA, prefrontal cortex, amygdala, and nucleus accumbens. The first maternal circuit was described in rats and is associated with a positive perception of the mother's baby and an instinct to care for the baby [55]. A decreased activity in the maternal circuit has been correlated with a withdrawal from caregiving [56]. Numan [55] found that the disruption of this circuit in the mPOA drastically reduces some aspects of maternal instincts, such as nest building. With the development of the fMRI in the early 2000s, a new method to investigate human neural plasticity arose [7],[57], and a human maternal circuit that involved the mPOA was identified [58]. The discovery of a similar circuit in many mammalian species revealed the mPOA's conserved role in coordinating parental care among mammals [58].

### 3.3. Pregnancy and postpartum-related volume changes in the maternal circuit

#### 3.3.1. Nucleus accumbens

While transient hypertrophy of the mPOA has been observed in female mice between pre-gestation and weaning [59], this phenomenon has not been observed in the human brain. In humans, other maternal circuit components change in volume during pregnancy and postpartum. For example, Hoekzema et al. [4] showed that the overall matter in the ventral striatum, the region of the brain that contains the nucleus accumbens, decreases in both the right and left hemispheres during pregnancy. This may indicate a reduction in the volume of the nucleus accumbens, which is a crucial part of the brain's motivation and reward system. Thus, a decrease in nucleus accumbens volume may indicate the “tuning” or adjustment of the female brain as she prepares to start caregiving for her child [60]–[62]. In addition, the volume reduction of the ventral striatum during pregnancy was positively associated with the strength of ventral striatum activation in response to offspring cues during the postpartum period [4]. Similarly, using resting-state fMRI data, Orchard et al. [63] recently found that both the parahippocampal gyrus and nucleus accumbens play significant roles in maternal caregiving by associating these regions with more efficient, flexible, and responsive behaviors toward infants.

Improper tuning of the maternal circuit may increase a mother's vulnerability to postpartum depression (PPD), which is a treatable condition that may be severely debilitating and is a common complication that arises from pregnancy and childbirth [64],[65]. For example, animal models that have structural reductions and functional changes in the nucleus accumbens, prefrontal cortex, and areas of the hippocampus have been observed to display PPD symptoms [66]–[68]. Lesions to the nucleus accumbens in animal models greatly hindered the development of a maternal experience effect [69].

#### 3.3.2. Amygdala

Parenting stimulates structural change to the amygdala of the maternal brain, which is another maternal circuit component. For example, Kim et al. [70] found an increase in grey matter in the maternal hypothalamus and amygdala, while Luders et al. [71] observed that structural changes of the amygdala were generated during the late postpartum stage. The maternal brain is exposed to elevated levels of estrogen during the peripartum period that impacts the amygdala. In rats, ERα expression decreased in the central nucleus and basal region of the amygdala during the gestational period that may increase the vulnerability of mothers to PPD [72]. As the amygdala is primarily responsible for emotional processing, these structural alterations and changes to hormone sensitivity may provide a neurobiological explanation for the drastic changes in a mother's emotions during pregnancy and, when dysregulated, contribute to PPD development.

The potential role of the amygdala in PPD development is supported by several MRI studies; for example, Ballesteros et al. [73] found that the volume of the right amygdala was reduced in mothers with PPD compared to healthy mothers. In addition, using resting-state fMRI, Mao [74] observed that the information flow between the amygdala and other parts of the brain, such as the frontal, occipital, and temporal lobes, was altered in women with PPD. Finally, using resting blood oxygen-level-dependent (BOLD) fMRI, Chase et al. [75] found a reduced connectivity between the amygdala and the posterior cingulate cortex (PCC) in women with PDD.

Future studies should investigate the neuroplasticity of specific, interconnected regions within the brain's mesolimbic reward system to pinpoint more precise mechanisms of PPD. Identifying a more specific neural mechanism in mothers could lead to the development of more effective treatments or prevention strategies. Future use of imaging technologies such as fMRI may be used to identify those at a higher risk of developing PDD by examining volume changes in the maternal circuitry, such as within the amygdala. In this manner, patients who would most likely benefit from treatment may be identified early in the postpartum period. Potential treatment strategies may focus on the administration of hormones such as oxytocin to alleviate PDD symptoms. Research into these possible lines of future treatment is particularly urgent considering the significant increase in PPD incidence, with reported rates more than doubling between 2010 and 2021 in one US study [76].

#### 3.3.3. Hippocampus

The hippocampus undergoes dramatic changes during pregnancy, the peripartum, and the postpartum periods. However, the hippocampus may either increase or decrease in volume, depending on the species examined and the timing of the measurement. For example, Wan et al. [77] observed increased hippocampal neurogenesis during pregnancy in the guinea pig that led to an increase in brain volume, while studies of mice [78],[79] and wild meadow voles [80] revealed reduced hippocampal neurogenesis during pregnancy. In addition, Hyer et al. [32] showed that hippocampal neurogenesis is promoted in an estrogen-dependent manner in mid-postpartum paternal California mice. Determining how pregnancy and the postpartum period impact the hippocampal structure is vital to understand the region's role in parental neurobiology, given that changes to the hippocampus may impact how parents manage stress. Farrar et al. [81] demonstrated that parental rock doves had elevated levels of glucocorticoid receptors expressed within their hippocampi. This might allow for the downregulation of the stress response through negative feedback control within the hypothalamic-pituitary-adrenal (HPA) axis to better enable parents to manage stress compared to non-parents [56]. Taken together, these results reveal that hippocampal neuroplasticity during pregnancy, the peripartum, and the postpartum period is not conserved across all mammalian species.

In human mothers, a reduction in the hippocampal grey matter volume in the early postpartum period (four months) is correlated with more positive caregiving [82]. The timing of these neuroplastic changes is dependent on the postpartum stage; for example, Hoekzema et al. [83] found that hippocampal volume decreases during pregnancy and early postpartum, then increases in the late postpartum stage. This contrasts with human fathers, where Saxbe et al. [84] found no significant change in the overall hippocampal volume between the pre- and postpartum periods. While the pattern of decrease-followed-by-increase in hippocampal grey matter volume is consistent in human mothers, no comparable changes were observed in human fathers.

### 3.4. Cortical grey and white matter volume changes within the maternal brain during postpartum

In addition to structural changes within specific subcortical brain regions, human mothers experience changes in the overall grey and white matter when becoming parents. For example, Pritschet et al. [85] tracked changes in the grey and white matter volumes of a healthy 38-year-old woman in a longitudinal study from preconception to two years postpartum using MRI. Pritschet et al. [85] found that the overall grey matter volume decreased and the white matter volume increased during this period. More recently, Servin-Barthet et al. [86] conducted a longitudinal study of 127 women who underwent their first pregnancy, 20 female partners of women in the pregnancy group, and 32 women without children and with no plans of going through pregnancy or parenting. In this study, Servin-Barthet et al. [86] acquired structural MRI scans and made endocrine determinations before conception, during pregnancy, and during the postpartum period at one and 6 months after birth. Servin-Barthet et al. [86] observed a “U-shaped” trajectory in the cortical grey matter volume, area, and thickness only among women who were pregnant, with a large grey matter reduction occurring during pregnancy followed by smaller increases in grey matter during the postnatal period. This “U-shaped” trajectory was associated with fluctuations in estrogen levels. Importantly, Servin-Barthet et al. [86] identified a positive correlation of postnatal grey matter volume recovery with increased mother-to-infant attachment, which is consistent with past work by Hoekzema et al. [4].

Kim et al. [87] performed a longitudinal study of 39 socioeconomically diverse, first-time mothers to examine the relationship between cortical thickness and the perceived adjustment to parenthood. In this study, Kim et al. [87] used MRI to identify increased cortical thickness in the superior frontal gyri, the lateral occipital gyri, and the inferior parietal gyrus during the first six months postpartum. Interestingly, Kim et al. [87] identified a positive association between cortical thickness in prefrontal regions and parental self-efficacy, thus revealing a role for changing prefrontal cortical thickness in the process of becoming a parent.

### 3.5. Changes within the human paternal brain

While many studies have revealed that significant reductions in the maternal gray matter volume occur during pregnancy [85],[86], a growing body of evidence suggests that experience-driven changes are also generated in the paternal brain. For example, Abraham et al. [88] used fMRI to reveal that primary-caregiving homosexual fathers demonstrate amygdala activation similar to that of primary-caregiving mothers. This result suggests that neuroplasticity in response to caregiving experiences may bridge neurological differences associated with maternal and paternal care. In addition, the experience of parenting impacts the organization of white and grey matter in the paternal brain. For instance, Cárdenas et al. [89] used diffusion-weighted imaging to measure white matter microstructural (WMM) organization in the entire brains of 38 fathers in a longitudinal study from prenatal to postpartum stages. Cárdenas et al. [89] observed decreases in WMM organization during the transition to fatherhood, with the largest WMM reductions occurring in the cingulum and the forceps minor in the corpus callosum. Additionally, Kim et al. [8] analyzed the grey matter of 16 fathers in a longitudinal study from 2–6 weeks to 12–16 weeks postpartum and observed grey matter volume reduction in the orbitofrontal cortex, posterior cingulate cortex, and insula and an increased grey matter volume in the hypothalamus, amygdala, striatum, and lateral prefrontal cortex. Increased grey matter volume in these regions may be important for fathers to connect with their young, as these regions are associated with emotional processing and regulation and motivation and reward. To learn more about the impact of parenting upon the paternal brain, we recommend further neurobiological research into paternal care in both heterosexual and homosexual parents, thereby investigating brain regions beyond the amygdala. While parental neurobiology has expanded to include more data on paternal care in recent decades, research on homosexual fathers and comparisons between parental brain function in heterosexual and homosexual parents remain limited [90]. Furthermore, there is a significant lack of data on the neurobiological adaptations experienced by foster care and adoptive parents, which warrants further investigation into these caregiving dynamics.

### 3.6. Structural changes in the parental brain persist into late adulthood

To examine the persistence of structural changes in the brain caused by parenthood into late adulthood, Orchard et al. [91] examined 573 adults aged 70–88 years. In a subsample of 235 healthy older women, a positive association between the number of children parented and cortical thickness in the parahippocampus, precuneus, cuneus, and pericalcarine sulcus was observed [91]. A positive association was also identified with the number of children parented and memory performance [91]. In addition, Orchard et al. [91] compared older non-parents to parents of one child in a sub-sample of 45 women and 35 men and found that 6 regions differed in cortical thickness in women and 5 regions differed in cortical thickness in men. Together, these results reveal some structural changes of the brain caused by parenting to persist into older age and can positively impact cognitive function. Structural changes in specific regions of the parental brain occur during both pregnancy and the postpartum period. Table 2 summarizes these changes in human mothers and fathers.

**Table 2.** Summary of maternal and paternal brain region volume changes associated with parenthood.

Parent	Brain Region	Matter Type Affected	Volume Change	References
Maternal and Paternal	Amygdala	Grey	Increase	Kim et al. 2014 [8]; Kim et al. 2010 [70]; Luders et al. 2021 [71]
Maternal and Paternal	Hypothalamus	Grey	Increase	Kim et al. 2014 [8]; Kim et al. 2010 [70]
Maternal	Hippocampus	Grey	Decrease then Increase	Hoekzema et al. 2022 [83]
Maternal	Nucleus Accumbens	Overall	Decrease	Hoekzema et al. 2020 [4]
Maternal	Substantia Nigra	Grey	Increase	Kim et al. 2010 [70]
Maternal	Grey Matter (Overall)	Grey	Decrease then Increase	Pritshet et al. 2024 [85]; Servin-Barthet et al. 2025 [86]
Maternal	White Matter (Overall)	White	Increase	Pritschet et al. 2024 [85]
Paternal	Corpus Callosum	White	Decrease	Cárdenas et al. 2024 [89]
Paternal	Insula	Grey	Decrease	Kim et al. 2014 [8]
Paternal	Lateral prefrontal cortex	Grey	Increase	Kim et al. 2014 [8]
Paternal	Orbitofrontal Cortex	Grey	Decrease	Kim et al.2014 [8]
Paternal	Posterior Cingulate Cortex	Grey	Decrease	Kim et al. 2014 [8]
Paternal	Striatum	Grey	Increase	Kim et al. 2014 [8]
Paternal	White Matter Microstructural (largest reduction in corpus callosum)	White	Decrease	Cárdenas et al. 2024 [89]

Note: Structural brain changes occur in maternal and paternal brains during pregnancy and postpartum. These changes can be categorized by the brain region affected, the type of tissue affected (white or gray matter), and the resulting change in volume.

### 3.7. Changes in the brain's structural components have functional consequences for parents

The transition to parenthood is associated with profound neurobiological adaptations in the brains of both mothers and fathers, thus driving behavioral changes essential for offspring care. The functional effects of these changes were first demonstrated in studies on rats in the early 2000s that established the link between structural alterations in the maternal brain and subsequent behavioral shifts [40],[92]. Neuroplasticity within the mPOA is crucial for maternal behaviors. For example, Keyser-Marcus et al. [92] found that mothers showed an altered mPOA plasticity in response to their pups during lactation, while Lee et al. [93] demonstrated that mPOA and amygdala lesions are correlated with a reduced operant rate for pup reinforcement in postpartum rats. Together, these results revealed a neuroanatomical basis for the motivational drive underlying maternal behaviors.

More recent neuroimaging technologies, such as fMRI, have enabled detailed functional observations of the human postpartum parental brain. In mothers, grey matter changes observed in the limbic system, temporal lobe, olfactory gyrus, and cerebellum, occurring at 6–12 weeks postpartum, are correlated with less hostile behaviors towards the newborn [94]. An fMRI study revealed that the grey matter changes during pregnancy are correlated with nesting behaviors, such as space preparation and selectivity [83]. This supports the idea that the brain undergoes significant structural reorganization, or neuroplasticity, that relates to behaviors preparing for the newborn's arrival [83].

Recent research into the neurobiology of parenthood confirmed that structural and functional brain changes differ between new mothers and fathers, thus strengthening the argument for functional behavioral differences in how mothers and fathers interact with their newborns. Atzil et al. [95] observed sex-based differences in neural activation among new parents: mothers showed higher amygdala activation, while fathers exhibited greater activation in socio-cognitive circuits. This may indicate functional differences in parental attachment styles depending on the parent's sex. Ho and Swain [96] demonstrated that primary caregiving mothers and fathers have similar activation levels within the limbic system and in socio-cognitive circuits. This indicates that the brain's functional activation is influenced by the parent's actual caregiving experience, thereby showing distinct neural adaptations based on whether they take on a primary or secondary caregiver role. Finally, reductions in the volume of and connectivity within the amygdala are correlated with PPD [73]–[75], heightened neural sensitivity to threatening infant cues, such as crying, and the impaired ability to associate newborn distress signals with healthy, nurturing parenting responses [96]. This further reveals how the dysregulation of amygdala function can influence parent-newborn interactions, contrasting the behavior of mothers with PPD with that of healthy mothers.

The impact of pregnancy and caregiving extends beyond immediate postnatal changes, as demonstrated by Orchard et al. [97], who revealed that having a greater number of children may prevent brain changes associated with aging. More specifically, Orchard et al. [97] demonstrated that older mothers and fathers who have multiple children better maintain functional connectivity across the brain. This preservation of connectivity could fundamentally alter aging trajectories in parents, potentially enabling those with more children to maintain a “younger brain” at an advanced age. While further investigation is essential, uncovering how a larger family size can help to prevent age-related brain decline in later life holds the potential for development of new treatments for neurological disorders in older adults.

Hormonal changes in the parental brain drive significant neuroplasticity in specific neural circuits and structures. These changes include both alterations to the white and gray matter volumes and remodeling within the limbic system. A key anatomical hub in this transformation is the mPOA, which is central to the conserved “parental circuit” across mammals. This evolutionary conservation underscores its fundamental adaptive purpose, thereby critically shaping the brain for the demanding functions of parenting offspring.

The limbic system is also integral to the “tuning” of the parental brain, with structural changes occurring in regions such as the amygdala, hypothalamus, and hippocampus. Throughout pregnancy and the postpartum period, the parental brain exhibits overall regional increases and decreases in both the gray and white matter. Importantly, while hormone fluctuations strongly correlate with many functional changes, these underlying structural alterations are also directly correlated with specific new parental behaviors that prepare the parent for the responsibilities of newborn care.

## 4. Conclusions

The mammalian brain undergoes vast changes in hormone levels and structural changes during pregnancy and postpartum. Work in rodent model systems has revealed that changes in estrogen, progesterone, and oxytocin signaling during pregnancy act on the mPOA to promote maternal nurturing behaviors. Further research should focus on the degree to which hormonal mechanisms of neuroplasticity are conserved across species, thereby paying special attention to the varying timescales upon which parental behaviors emerge. Moreover, the field of parental neuroscience requires efforts to standardize the quantification of parental behaviors, as critical variables are currently inconsistently measured across studies. For instance, developing a standard index for key behaviors—such as the latency to pup retrieval (gathering), licking and grooming, and pup huddling—would significantly improve the reliability and comparability of data across different laboratories.

Human research has revealed that hormonal changes during pregnancy impact components of the maternal circuit, including the amygdala, hypothalamus, hippocampus, and other reward pathways of the brain. Research into the hormonal and neural mechanisms which underlie fatherhood and foster parenthood is a much younger field and currently lacks the cohesion seen in maternal studies. In addition, hormonal changes occur during the postpartum in diverse caregivers to establish, rather than to maintain, parental behaviors, and these changes can cause alterations in the brain volume. Exactly what nurturing behaviors are impacted by changes to which brain structures during pregnancy and the postpartum is a question currently undergoing active investigation. Future study should reveal more detail about the changes that are generated in the brain during parenthood and how these alterations impact caregiving behaviors. Therefore, we will gain more insight into how best to help parents when these changes are dysregulated or form atypically, including among parents who have PDD.

## Use of AI tools declaration

The authors declare they have not used Artificial Intelligence (AI) tools in the creation of this article.
